# The Health of the School Child

**Published:** 1922-11

**Authors:** 


### THE HEALTH OF THE SCHOOL CHILD.

THE report for 1921 by Sir George Newman, Chief Medical Officer to the Board of Education, shows that, though many children's ailments appear to be trifling, they may be the beginning of serious diseases. The child is inspected, and, if found defective, is referred for treatment, is " followed up," and is subsequently re-inspected. The inspection accords with a recognised standard laid down by the Board in 1908, and takes place in its routine form at least thrice during the nine years of the child's school life—on admission, at the age of 8, and before leaving school—as well as at other times when necessary. This means an annual medical examination of 2,500,000 children. To carry out this work each authority has appointed a school medical officer, and, where necessary, assistant medical officers. The total number of medical men engaged (whole or part time), including specialists for consultation, is now approximately 2,000. There are also some 3,000 school nurses, partly or wholly occupied in school work. Experience rapidly showed that much more was necessary, particularly on the preventive side, than mere medical inspection and treatment in the narrow sense. Gross uncleanliness was found prevalent in many areas, and cleansing schemes, school baths, and periodical surveys proved necessary. Malnutrition lies at the bottom of much poor physique, and the school doctor recommends food for malnourished necessitous children under the Provision of Meals Act of 1906. Some of the malnutrition and much of the physical impairment were found due to lack of proper exercise, and the Board introduced a new system of physical training for all schools. Anæmia and other diseases are due to lack of sunlight and fresh air, in themselves of high therapeutic value, and open-air schools were introduced. The defective children, the blind, the deaf, the feeble-minded and the cripples were referred increasingly to the Special Schools, where specialised training is provided. The little children entering school for the first time were found to be suffering from physical defects to the extent of 30 or 40 per cent., and the Nursery School principle was formulated. All children of school age in all classes were found to be in great need of instruction in the knowledge and practise of the laws of hygiene, and a scheme of teaching was devised for all schools.

Some 10 per cent. of the children in attendance at public elementary schools, though not mentally deficient, proved to be seriously dull and backward and unable to take advantage of the ordinary education provided. For these children special classes have been arranged. Likewise the child suffering from tuberculosis could not be dealt with in school, and for such children accommodation had to be found in sanatorium schools, where both treatment and education are supplied.

Lastly, as the children grew older, they had to be "followed up" in schools of higher education, and there emerged the large and far-reaching question of employment, both during school life and subsequently. It is an old story in English history how the health of the child has been sacrificed to industry, and it is not the least of the developments of school hygiene that this year child labour by means of school exemption will universally become illegal under the Education Act of 1918.

Of the total expenditure of Local Education Authorities, the Exchequer pays to the authorities approximately half in the form of grant in aid, the other half falling on the local rates. Although this expenditure is heavy, it was the considered view of the Geddes Committee that curtailment would be a disadvantage to the community.

In 1920 to 1921 the total expenditure was as follows:—

| | |
|---|---|
| School Medical Inspection and Treatment | £1,330,182 |
| Special Schools | 1,253,105 |
| Physical Training | 39,872 |
| Evening Play Centres | 95,513 |
| Nursery Schools | 11,335 |
| Provision of Meals | 252,891 |
| | £2,982,898 |

It is nevertheless clear that certain local authorities might well exercise more supervision of costs, in view of the national situation. The school medical service on the average costs, per child attending a public elementary school, 5s. in boroughs and 6s. for urban districts, but in one borough it is as low as 1s. per child, and in one urban district as high as 21s. per child. Similarly, the cost of meals varies unduly. In Leicester the average total cost per meal is 10½d., and in Dudley less than 2d. Sir George Newman points out that in many areas children have been admitted to free meals whose family circumstances neither necessitated nor justified this. In certain areas the local administration has succeeded in transferring a substantial part of the burden of poor relief to the education rate, and expenditure has been incurred in the provision of meals on a scale quite outside the scope intended by Parliament.

### HEALTH WEEK.

HEALTH Week is intended to lead the less advanced parts of our population to connect the idea of health with personal and domestic hygiene rather than with what they call "doctors' stuff." Its promoters impress upon those who arrange local celebrations that their aim should be to arouse a sense of personal responsibility; to emphasise the value, in terms of happiness, of cleanly habits and surroundings, and not to pander to morbid tastes with gruesome details of disease. Useful educational work was done in many parts of the country during the period Oct. 8-14. At York, which has held a Health Week for the first time this year, the leading idea was admirably carried out by the lecturers. Professor Andrew Allison, Professor of Jurisprudence and Public Health at St. Mary's College, Glasgow, spoke on "The Responsibility of the Individual and the State in the Prevention of Disease." He pointed out that it did not matter how pure milk was when delivered, if in the house it was exposed to dust and flies; that the State could enforce the building of suitable houses, but could not ensure that such houses were properly used; that the loss of infant life due to measles was to a large extent avoidable if people would only recognise that it was not a trivial disease, and would protect their children from infection and give them prompt and adequate treatment. Dr. W. E. Collinge, Keeper of the Yorkshire Museum, in speaking on flies and other household pests, advocated the compulsory provision by builders of private houses of proper arrangements for keeping food. Other lecturers urged the need for exercise, especially walking, and for the revival of the art of domestic cookery and greater care in the selection of food.

### HEALTH LECTURES IN PRISONS.

THE People's League of Health has made an extraordinary new departure by extending its propaganda to the gaol. By arrangement with the Home Office, lectures on "Health of mind and body, how to obtain and preserve it," are being delivered by members of the medical council of the League in the London prisons. During the autumn and winter they will be extended to other prisons throughout the country. The League is to be commended for its new effort. Criminals are a class prone to offend against the laws of hygiene, as they are against other laws, and those who are reclaimable are likely to profit by the instruction. Moreover, the knowledge that such an interest is taken in their welfare must have a good effect and help to reclaim them.

